# The Fly Muscles in on Glycogen Autophagy

**DOI:** 10.1371/journal.pbio.1001709

**Published:** 2013-11-12

**Authors:** Caitlin Sedwick

**Affiliations:** Freelance Science Writer, San Diego, California, United States of America

[Fig pbio-1001709-g001]When faced with starvation, cells can recycle proteins and organelles to recapture energy and resources through a process called autophagy. During autophagy, double-layered membrane vesicles called autophagosomes encircle cytoplasmic components. Autophagosomes then fuse with the cell's digestive organelle (the lysosome) so their contents can be broken down. This can be critical for cell survival through lean times.

**Figure pbio-1001709-g001:**
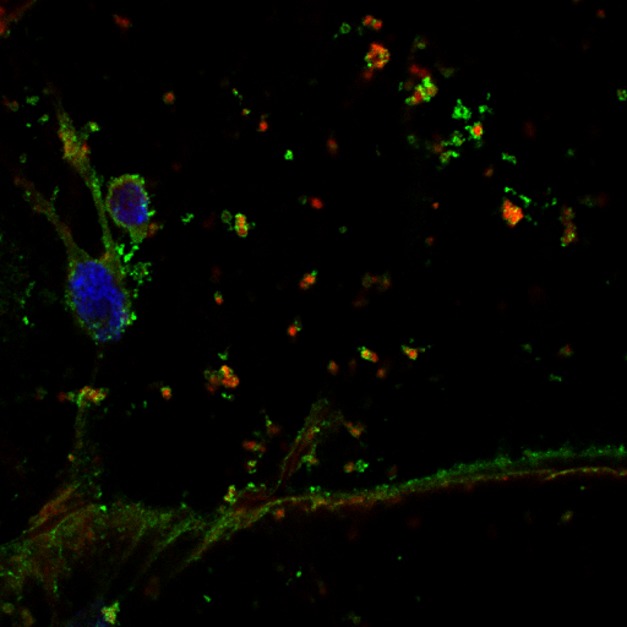
Fruitfly larvae fed with the drug chloroquine accumulate glycogen-filled vesicles in their skeletal muscles. Glycogen synthase (green) and the core autophagy component, Atg8 (red), localize to these vesicles, which deliver glycogen to the lysosome in response to starvation. Nuclei are stained in blue.

If autophagy is impaired, it can have serious consequences. For example, fast-twitch skeletal muscle cells contain large stores of energy in the form of glycogen that can be diverted for breakdown by the autophagy pathway in case of starvation. But there are several human diseases in which glycogen accumulates in muscle autophagosomes. These autophagosomes become so bloated with glycogen that they displace and damage the contractile fibers of the muscle cell, resulting in myopathy. Many glycogen-related myopathies have genetic causes, but they can also appear in people treated with the anti-malarial drug chloroquine (CQ), which prevents autophagosomes from fusing with the lysosome. Unfortunately, it is difficult to study many of these diseases because it isn't known how glycogen normally reaches the autophagic pathway in the first place. That's what inspired Jonathan Zirin, Joppe Nieuwenheus, and Norbert Perrimon to take a closer look at the autophagic breakdown of glycogen in their paper published in this month's *PLOS Biology*.

To determine which genes and proteins are involved in autophagic glycogen degradation, Zirin and colleagues turned to the fly *Drosophila melanogaster*. The muscle of fly larvae, the group showed, is a good system in which to study autophagy because its cells form autophagosomes when the animals are deprived of nutrients. Immunofluorescence and electron microscopy demonstrated that autophagosomes in starved larval muscle contain glycogen, and that glycogen is the main target for autophagy in these cells. Consistent with the idea that glycogen is broken down via autophagy, glycogen levels progressively decline over time in starved larval muscle.

Significantly, Zirin et al. found that larvae treated with CQ exhibit signs of myopathy and motor dysfunction just as in humans. In starved animals treated with CQ, muscle cells contain many extra, glycogen-containing autophagosomes. And, just as in humans, these autophagosomes fail to fuse with lysosomes, and eventually become so bloated that they disrupt muscle contractile fibers. Meanwhile, CQ treatment also slows down the degradation of glycogen in starving muscle. It's likely this is due to CQ's disruption of the autophagy pathway because glycogen degradation is impaired to a similar degree in cells lacking expression of proteins critical for autophagy. Nonetheless, neither CQ treatment nor loss of autophagy pathway proteins can completely stop the glycogen breakdown during starvation, suggesting there are other pathways the cells can use to degrade glycogen. In fact, the authors found that an enzyme called glycogen phosphorylase operates independently of the autophagy pathway to break down glycogen in starving larval muscle.

Having traced the fate of glycogen in starving cells, the authors were curious about how cellular glycogen levels might affect the autophagy pathway. To look at this question, they used RNAi to knock down expression of the gene encoding the fly enzyme that makes glycogen, *GlyS*. As expected, loss of GlyS protein caused cells to have lower baseline glycogen levels. But, it didn't affect the cells' ability to form autophagosomes upon starvation; just as many autophagosomes form in GlyS-deficient muscle as are found in normal muscle. The main difference is that the autophagosomes of GlyS-deficient animals don't become bloated after CQ treatment like the autophagosomes of normal animals do. This points to an important role for GlyS in CQ-induced autophagosome bloating.

How is GlyS involved in CQ-induced autophagosome bloating? It is possible that bloated autophagosomes simply can't occur in GlyS-deficient cells because the cells have such a low baseline level of glycogen. However, there is another possibility: GlyS may be responsible for bringing glycogen to autophagosomes in the first place. This idea is supported by the authors' demonstration that GlyS binds to an autophagosomal protein called Atg8. By introducing various mutations into GlyS, the researchers showed that this interaction only takes place during starvation and that it is enhanced when GlyS is enzymatically active. Therefore, during starvation, GlyS may simultaneously bind to both Atg8 and glycogen, thereby bringing glycogen to the autophagosome. If that's the case, then autophagosomes could become bloated if GlyS continues this activity even when (as happens with CQ treatment) autophagosomes cannot unload their cargo at lysosomes.

Altogether, these data provide useful new insights into both glycogen autophagy and CQ-induced myopathies. And this is just the beginning of what we can learn using this new fly model for glycogen autophagy.

**Zirin J, Nieuwenhuis J, Perrimon N (2013) Role of Autophagy in Glycogen Breakdown and Its Relevance to Chloroquine Myopathy.** doi:10.1371/journal.pbio.1001708

